# Decomposition of abnormal free locomotor behavior in a rat model of Parkinson's disease

**DOI:** 10.3389/fnsys.2013.00095

**Published:** 2013-11-27

**Authors:** Benjamin Grieb, Constantin von Nicolai, Gerhard Engler, Andrew Sharott, Ismini Papageorgiou, Wolfgang Hamel, Andreas K. Engel, Christian K. Moll

**Affiliations:** ^1^Department of Neurophysiology and Pathophysiology, University Medical Center Hamburg-Eppendorf, University of HamburgHamburg, Germany; ^2^Department of General Psychiatry, Center for Psychosocial Medicine, University of HeidelbergHeidelberg, Germany; ^3^Centre for Integrative Neuroscience, University of TübingenTübingen, Germany; ^4^Medical Research Council, Anatomical Neuropharacology Unit, Department of Pharmacology, University of OxfordOxford, UK; ^5^Division of General Neurophysiology, Institute of Physiology and Pathophysiology, University of HeidelbergHeidelberg, Germany; ^6^Department of Neurosurgery, University Medical Center Hamburg-Eppendorf, University of HamburgHamburg, Germany

**Keywords:** 6-OHDA lesions, stereology, spontaneous activity, Parkinson disease, video monitoring

## Abstract

Poverty of spontaneous movement, slowed execution and reduced amplitudes of movement (akinesia, brady- and hypokinesia) are cardinal motor manifestations of Parkinson's disease that can be modeled in experimental animals by brain lesions affecting midbrain dopaminergic neurons. Most behavioral investigations in experimental parkinsonism have employed short-term observation windows to assess motor impairments. We postulated that an analysis of longer-term free exploratory behavior could provide further insights into the complex fine structure of altered locomotor activity in parkinsonian animals. To this end, we video-monitored 23 h of free locomotor behavior and extracted several behavioral measures before and after the expression of a severe parkinsonian phenotype following bilateral 6-hydroxydopamine (6-OHDA) lesions of the rat dopaminergic substantia nigra. Unbiased stereological cell counting verified the degree of midbrain tyrosine hydroxylase positive cell loss in the substantia nigra and ventral tegmental area. In line with previous reports, overall covered distance and maximal motion speed of lesioned animals were found to be significantly reduced compared to controls. Before lesion surgery, exploratory rat behavior exhibited a bimodal distribution of maximal speed values obtained for single movement episodes, corresponding to a “first” and “second gear” of motion. 6-OHDA injections significantly reduced the incidence of second gear motion episodes and also resulted in an abnormal prolongation of these fast motion events. Likewise, the spatial spread of such episodes was increased in 6-OHDA rats. The increase in curvature of motion tracks was increased in both lesioned and control animals. We conclude that the discrimination of distinct modes of motion by statistical decomposition of longer-term spontaneous locomotion provides useful insights into the fine structure of fluctuating motor functions in a rat analog of Parkinson's disease.

## Introduction

Neurotoxin-induced degeneration of nigral dopamine neurons in experimental animals results in motor abnormalities relevant to motor symptoms of Parkinson's disease (PD; Cenci et al., 2002). One strategy to deplete midbrain dopaminergic neurons in rats is to infuse the neurotoxin 6-hydroxydopamine (6-OHDA) directly into the substantia nigra pars compacta (SNc; Schwarting and Huston, 1996a,b), which can also cause moderate cell death in the neighboring ventral tegmental area (VTA). In the prototypic toxin-induced rat model of PD, unilateral intracerebral 6-OHDA injections lead to the expression of a strictly lateralized hemiparkinsonian phenotype, the behavioral sequelae of which have been described in great detail. A wide variety of different behavioral tests are used to examine motor changes associated with the asymmetrical depletion of the nigrostriatal dopaminergic system, e.g., skilled motor tasks (Mokrý et al., 1995; Truong et al., 2006), footprint-analysis (Metz et al., 2005), treadmill running (Brazhnik et al., 2012), or drug-induced rotation (Ungerstedt, 1971; Kelly, 1975). Compared to the unilateral 6-OHDA model, bilaterally lesioned rats have been used less commonly, although they display a far more severe parkinsonian phenotype. In this respect, the slowness and scarcity of movement observed in the bilateral 6-OHDA rat model resembles more closely the marked expression of PD-like symptoms in monkeys treated systemically with 1-methyl-4-phenyl-1,2,3,6-tetrahydropyridine (Bergman et al., 1990) and cardinal motor manifestations of human PD patients in advanced disease stages. The development of pronounced motor impairment in rats with severe bilateral 6-OHDA lesions is, however, often accompanied by aphagia, adipsia and abulia (Schallert et al., 1978; Sakai and Gash, 1994; Cass et al., 2005; Ferro et al., 2005).

Rat equivalents of akinesia are commonly assessed in an “open-field” environment. Most often, rat motion (including scanning, walking or running, as well as rearing) is detected by consecutively breaking light beams arranged in an array around the arena (Cass et al., 2005; Ferro et al., 2005; Belujon et al., 2007). Other methods to quantify locomotor capacities are wheel (Schallert et al., 1978) or treadmill running (Avila et al., 2010). These standard measurements usually assess a short period of 10–60 min of locomotor activity (Cass et al., 2005; Ferro et al., 2005; Belujon et al., 2007), assuming a stable motor phenotype over time. However, these methods may underestimate the complexity of exploratory behavior, in particular long-term motor fluctuations.

Recently, a novel analytical approach to video-based tracking data utilized the statistical discrimination of rat motor behavior on the basis of speeds (Drai et al., 2000; Drai and Golani, 2001). This form of data analysis showed that naïve rats, but also naïve mice (Drai et al., 2001), use distinctly different modes of motion to explore their environment. The current study aimed to assess the impact of dopamine depletion on these naturally occurring behavioral patterns. We hypothesized that expression of a severe parkinsonian phenotype would not only reduce overall motion speed, as could be assessed with other standard quantification methods, but would also alter the fine spatio-temporal structure of exploratory locomotion modes. To test this, we adapted the analysis approach of Drai and colleagues to video-based tracking data from 23 hours of continuous and spontaneous locomotion of bilaterally 6-OHDA lesioned and control rats.

## Materials and methods

### Animals

Animal experiments were approved by the local government authorities of Hamburg and carried out in accordance with the European Council Directive 86/609/EEC. All experiments were performed on male Brown Norway rats (Rattus norvegicus; Charles River Laboratories, Sulzfeld, Germany). All efforts were made to minimize suffering. The bilateral 6-OHDA lesion model is known to be associated with high mortality due to aphagia/adipsia and following loss of body weight (Ferro et al., 2005). Careful clinical inspection and weighting of animals was performed on a daily basis. In general, we offered a soft and moist rat chow and 10%-glucose solution in addition to standard chow and water *ad libitum*. To sustain aphagic and adipsic PD rats, we administered a liquid and high caloric nutrition for rodents (Altromin, Lage, Germany) by manual needle feeding. In total, the dropout rate was 5/14 PD rats. Dropouts included perioperative death (*n* = 2 PD) and loss of >20% body weight without stabilization of weight loss within 14 days after surgery (*n* = 3 PD) which led to euthanasia of these animals by decapitation under deep anesthesia (i.p.-injection of ketamine 100 mg/kg, xylazine 6 mg/kg).

### Experimental design

We randomly assigned 14 rats to a PD group receiving bilateral 6-OHDA injections into the SNc and eight rats to a control group receiving bilateral vehicle injections (preoperative weight: 384 ± 32 g, mean ± *SD*). Video tracking of 23 h of spontaneous locomotor activity was performed prior to surgery. Due to the labor intensity of sustaining PD rats we ran the experiment in two phases. Within the first subset of 10 rats (*n* = 8 PD; *n* = 2 controls) the dropout rate was 4/8 PD rats. The second subset of 12 rats (*n* = 6 PD; *n* = 6 controls) exhibited a dropout rate of 1/6 PD rats. Postoperative locomotor activity was assessed after a recovery period of 12 ± 2 days in the first subset and 28 ± 2 days in the second subset. After postoperative video monitoring, all rats were subsequently used in a separate study. We sacrificed rats after induction of deep anesthesia (ketamine/xylazine) ~14 weeks after lesioning by transcardial perfusion with saline. A midbrain tissue block was immersion-fixed in 4%-PFA-solution (paraformaldehyde in 0.1 M phosphate buffered saline, Sigma-Aldrich) for subsequent histological TH-staining and stereological counting of tyrosine hydroxylase (TH)-positive SNc and VTA neurons.

### Bilateral 6-hydroxydopamine lesions

Stereotactic injections were performed under general anesthesia introduced with isoflurane (Baxter Germany GmbH, Unterschleißheim, Germany) and maintained with i.p.-injections of ketamine (65 mg/kg, Dr. E. Gräub AG, Bern, Switzerland) and xylazine (3 mg/kg, Bayer Health Care, Leverkusen, Germany). Thirty minutes prior to 6-OHDA or vehicle injections, rats received a bolus i.p.-injection of desipramine (25 mg/kg, Sigma-Aldrich, Munich, Germany) to minimize uptake of 6-OHDA in noradrenergic midbrain neurons (Schwarting and Huston, 1996b). Cardiopulmonary protection was assured by an initial bolus injection of atropine (0.25 mg/kg, B. Braun Melsungen AG, Melsungen, Germany). Body temperature was monitored during surgery with a rectal probe and hypothermia was prevented with an adjustable heating pad (FST, Heidelberg, Germany). In addition, the eyes were covered with dexpanthenol cream to prevent exsiccation. Animals were mounted in a stereotactic frame (David Kopf Instruments, Tujunga, USA). To target the SNc a Hamilton microliter-syringe (FST) was lowered through a burr hole placed at +4 mm AP and ±2.2 mm ML using the interaural line as reference (Paxinos and Watson, 2005). The syringe was slowly lowered to the target depth at −8 mm relative to the dura. Five microliter neurotoxin (3 μg/μl 6-OHDA hydrochloride free base in 0.2% ascorbic acid solution, stored on ice; Sigma-Aldrich) or vehicle (aqua injectabilia, 0.2% ascorbic acid solution; Sigma-Aldrich) was slowly infused at a rate of 0.5 μl/min and the syringe was left in place for 2 min to allow for complete absorption of the toxin. The burr hole was closed with bone wax and the procedure was repeated in the contralateral hemisphere. Rats received s.c.-injections of metamizole (100 mg/kg, Medistar, Holzwickede, Germany) for postoperative analgesia following surgery and on behavioral signs of pain distress.

### Stereology

**Figure 1:**
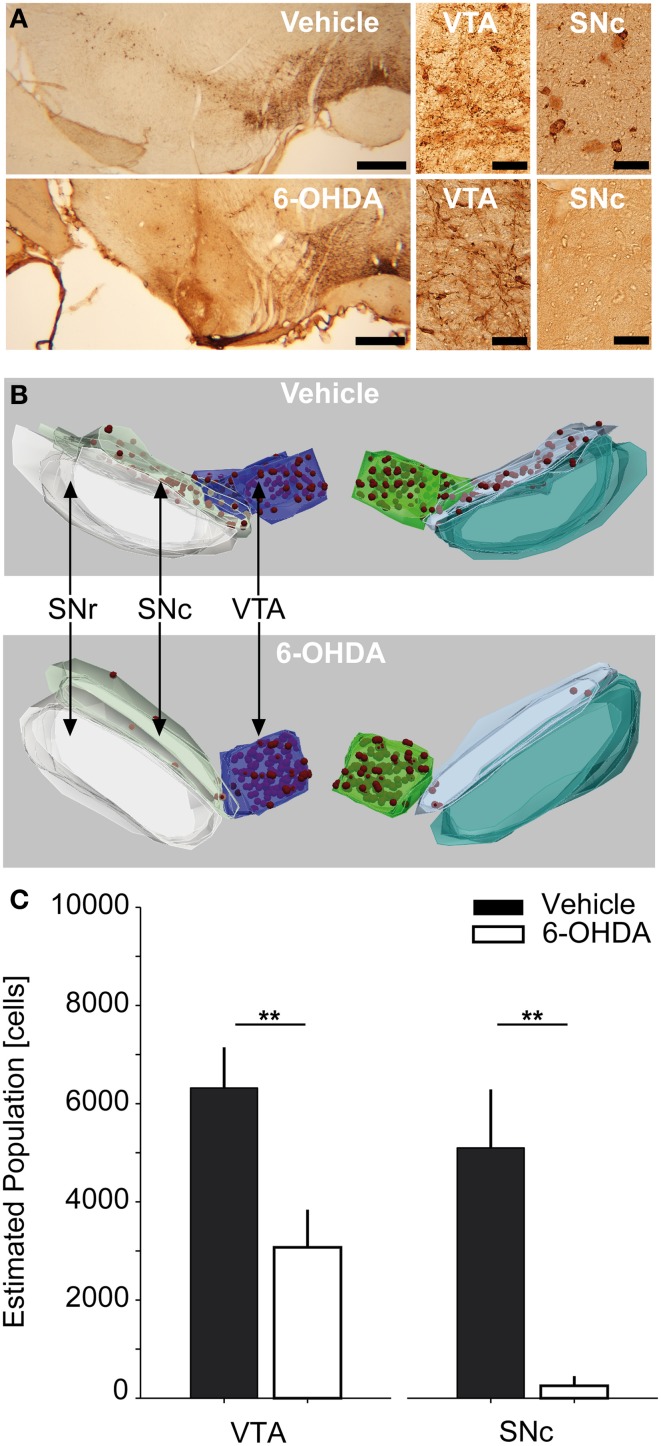
**Stereological counting of TH-positive SNc and VTA neurons. (A)** Representative photomicrographs of TH-stained midbrain sections of a vehicle and 6-OHDA injected rat (left column, scale bar equals 500 μm) as well as 40× magnifications of VTA and SNc-ROIs (middle and right column, scale bar equals 50 μm). **(B)** 3D-reconstructions of stereological regions of interest for the same vehicle and 6-OHDA injected rats as in **(A)**. Red spheres indicate stereologically counted cells in the SNc and VTA. **(C)** Estimated absolute population of cells in the SNc and VTA for vehicle (*n* = 5) and 6-OHDA (*n* = 8) injected rats. Double asterisks denote *P* ≤ 0.001 (uncorrected). TH, tyrosine-hydroxylase; 6-OHDA, 6-hydroxydopamine; VTA, ventral tegmental area; SNc, substantia nigra pars compacta; SNr, substantia nigra pars reticulata.

To count TH-positive dopaminergic cells in PD rats and controls we performed free-floating TH-immunohistochemistry on serial 40 μm coronal sections of the midbrain. Figure 1A displays representative examples of photomicrographs depicting TH-stained midbrain sections from a vehicle and 6-OHDA injected rat. Due to technical reasons we could only obtain stereology in a subset of 8/9 PD-rats and 5/8 controls. PFA-fixed tissue blocks containing SNc and VTA were transferred to 30%-sucrose solution and kept at 4°C for 24 hours. Sections were cut with a freezing-microtome (Leica Instruments, Wetzlar, Germany) and stained in free-floating fashion for TH-activity. Briefly, sections were washed in phosphate buffer (0.01 M PBS, Sigma-Aldrich), incubated with 3%-H_2_O_2_-solution for 3 min to block endogenous peroxidase activity and incubated with 2% normal horse serum (added with 0.3% Triton X-100, Sigma-Aldrich) for 30 min. Sections were then incubated over night at 4°C with the primary TH-antibody (1:250, monoclonal mouse antibody, Novocastra reagents, Leica Microsystems, Wetzlar, Germany), followed by the biotinylated secondary antibody (1:400, Novocastra reagents) for 30 min, and afterwards incubated with avidin and biotinylated horseradish peroxidase (ABC kit, Novocastra reagents) for another 30 min. TH was visualized by adding peroxidase substrate (0.02% DAB reagent in 0.003% H_2_O_2_ in PBS) for 2–10 min duration. Finally, sections were mounted on glass slides, dehydrated in an increasing alcohol row and fixed under a cover slid with Roti Histokitt II (Carl Roth, Karlsruhe, Germany).

Unbiased stereological counting of TH-positive cells in the SNc and VTA was performed using the Stereoinvestigator Software (Version 10.0, MicroBrightField Inc., Williston, Vermont, USA). The hardware consisted of an Olympus Bx61 brightfield microscope (Olympus Deutschland GmbH, Hamburg, Germany) equipped with a Microfire TM A/R camera (Optronics, California, USA) and an x-y-z galvano table (Carl Zeiss AG, Jena, Germany). The optical fractionator probe (West et al., 1991; West, 2002) was applied on series of 40 μm thick coronal sections. To assure sampling of comparable structural parts, the stereological analysis was centered on an independent anatomical hallmark (the rootlets of the oculomotor cranial nerve). Overall we sampled 10 sections (sampling rate of 1.2 ± 0.3 mean ± *SD* sections) spanning ~480 μm in the cranio-caudal dimension. However, due to availability of continuous TH-sections containing SNc and VTA, three rats were investigated using 7, 8, or 9 sections, respectively. Using a 2.5× magnification with numerical aperture of 0.075 we defined three anatomical regions of interest (ROI), i.e., the SNc, the VTA and the substantia nigra pars reticulata (SNr) (Paxinos and Watson, 2005). For examples of anatomical 3-D reconstructions of ROIs and counted cells, see Figure 1B. Cell counting was performed using a 40× Plan-Neofluar dry type objective lens with a numerical aperture of 0.75 (Carl Zeiss AG) within the SNc and VTA-ROIs. The counting frame (50 × 50 μm) with dissector height of 20 μm was applied with a uniform random sampling grid of 150 × 150 μm (optical dissector volume of 50,000 μm^3^, sampling grid area of 22,500 μm^2^, Gundersen, 1986). Schaeffer's estimated coefficient of error ranged between 0.05 and 0.12 for the VTA of both controls and PD animals, as well as the SNc of controls. For SNc-ROIs of PD animals it ranged between 0.3 and 0.9, reflecting the scarceness of TH-positive cells in this area (Gundersen, 1986; West, 2002). The total volume of the sampled SNc and VTA parts was stereologically estimated using the Cavalieri method (Gundersen and Jensen, 1987). Our histological regime resulted in robust sampling >200 neurons per VTA and SNc in vehicle injected controls, enabling us to express TH-positive cell numbers in absolute numbers. Cell counts were calculated separately for each ROI and hemisphere, respectively, and combined across hemispheres (*n* = 6 hemispheres per group).

To assess the symmetry of depletion we calculated the laterality index *(LI)* for each rat (Seghier, 2008), *LI* = *f* × (*E*_*LH*_ − *E*_*RH*_)/(*E*_*LH*_ + *E*_*RH*_), where *E*_*LH*_ and *E*_*RH*_ are the combined VTA and SNc estimates of TH-positive cells for the left and right hemisphere, respectively, and *f* is a scaling factor set to 1 resulting in the *LI* to be bound between −1 and 1.

### Behavioral monitoring

Locomotion of 6-OHDA or vehicle injected rats was investigated via continuous video monitoring of 23 hours of spontaneous behavior in an open field-like environment. We used an infrared video-based tracking system (VideoMot 2.0, TSE Systems, Bad Homburg, Germany) to record movement paths as time series of x-y-positions at a sampling rate of 12.8 Hz. The recording arena was spaced 70 × 100 cm with 40 cm high walls (i.e., 320 × 435 pixel after frame grabbing and offline analysis) and equipped with three food pellet feeders and one water outlet positioned in the corners. The ground was covered with bedding. The arena size was chosen to be ~3 times larger than the size of the rat's home cage to allow for generation of more naturalistic movement patterns including running. The arena was placed in a custom-made recording box lined with foamed plastic to ensure light and acoustic insulation. Recordings took place under constant darkness.

### Behavioral data analysis

**Figure 2:**
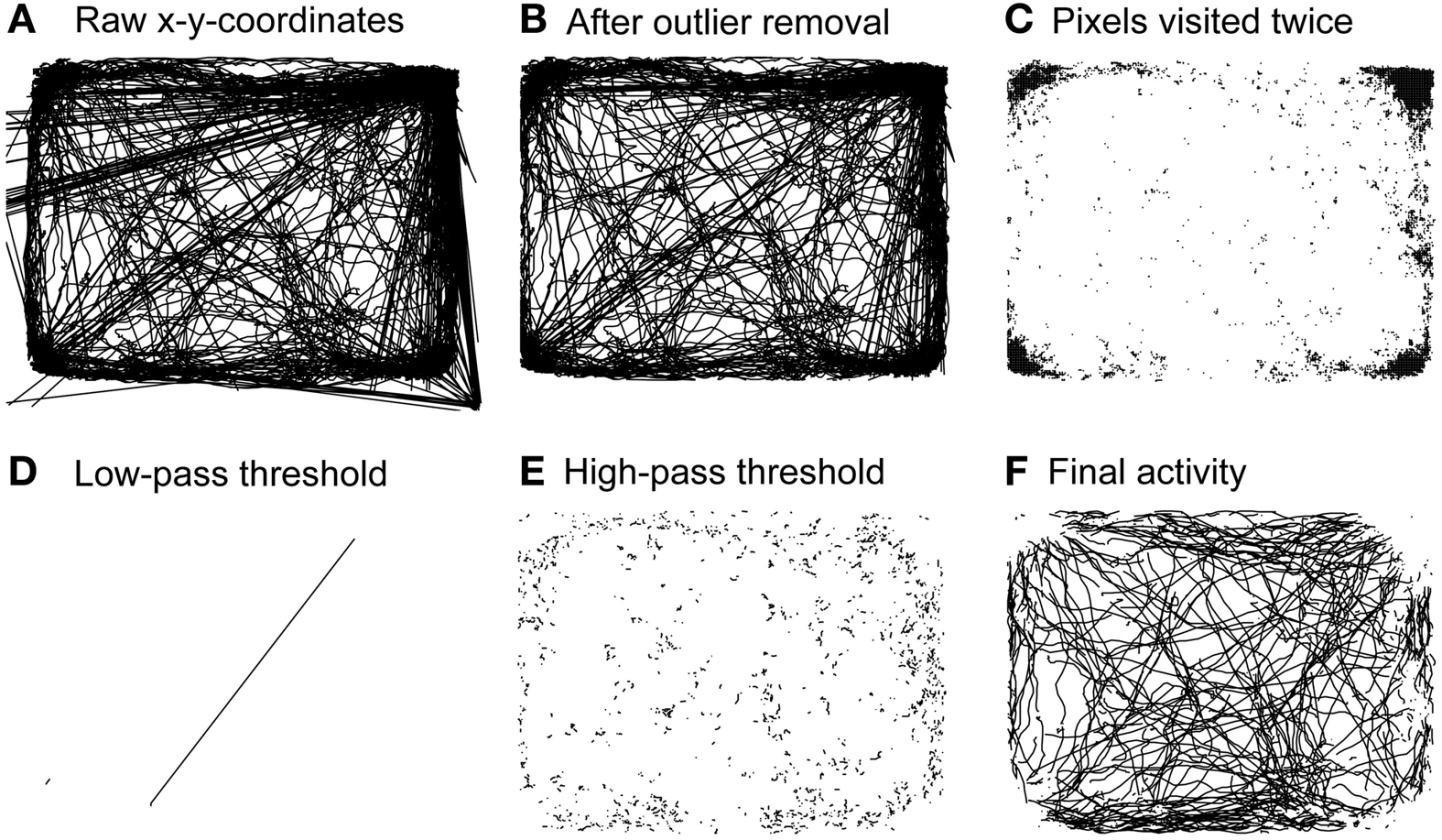
**Steps of behavioral analysis**. Activity of a representative rat during 23 h of pre-operative monitoring. **(A)** Raw activity including all artifacts. **(B)** Activity remaining after outlier rejection. **(C)** Spaces on the single pixel level that were “visited” twice during the whole recording time. **(D)** Artifacts excluded through application of a low-pass threshold of 80 cm/s. **(E)** Resting activity excluded through application of a high-pass threshold of 4 cm/s. **(F)** Activity used for subsequent behavioral analyses.

To analyze spontaneous long-term behavior we modified a data processing and analysis approach developed for open-field locomotion in rodents (Drai et al., 2000; Drai and Golani, 2001). All routines were written in MATLAB (The Mathworks, Natick, USA). The analytical approach is based on the idea of calculating single speed values (cm/s) from x-y-position time series using a sliding window, separating rest vs. motion episodes and characterizing motion episodes by the maximal speed reached within each episode rather than the average speed of a given episode. Our modified analysis consisted of five separate analysis steps (see Figure 2): First, raw x-y-position time series data obtained for 23 hours at a sampling rate of 12.8 Hz (~1,000,000 single data points) contained large amounts of artifacts (see Figure 2A). Typically, artifacts resulted from spurious “jumping” within a continuous x-y-position time series to distant places and back. Frequent sources of artifacts lay outside the actual recording arena and could therefore be removed through spatial outlier rejection. However, artifacts also appeared within the arena, generally jumping toward the arena's corners where animals spent most of their resting time (Figure 2B). All places on the pixel level that were “visited” twice during the recording time were neglected, eliminating artifacts associated with resting spots (Figure 2C). To further de-noise the recordings we applied a low-pass threshold of 80 cm/s (Figure 2D), as preliminary data screening showed that no rat reached speed levels >70 cm/s inside the recording arena (data not shown). Single speed values were calculated on position time series with a moving window of 0.3 s (Drai et al., 2000; Drai and Golani, 2001). Separation of resting vs. motion episodes was done by high-pass thresholding speed values at a noise level of 4 cm/s (Figure 2E). This threshold was based on previous work of Drai et al. (2000) and adapted to the distribution of single speeds in our data set with a peak at 4 cm/s (data not shown). The remaining motion episodes were used for further analysis (Figure 2F). To compensate for erroneous separation of movement episodes resulting from artifact correction we interpolated x-y-coordinates of single missing values.

### Behavioral endpoints

Smoothed histograms of log transformed maximal speed values, termed “log max-SD,” were used to identify different modes of motion in control and PD rats (Drai et al., 2000; Drai and Golani, 2001). A Gaussian mixture model was fitted toward the empirical data to obtain information about the localization of different “gears” of motion within the distribution of speeds. Gears were separated at 10 cm/s, a value consistently derived from preoperative recordings. Descriptive locomotion parameters were calculated for slow (i.e., first gear) and fast (i.e., second gear) episodes. However, as the dissection of second gear episodes allowed a view on full-blown motion, we concentrated our analysis on such episodes.

The six different behavioral endpoints were defined as: (1) incidence of fast speed movement episodes, which is the percentage of second gear episodes relative to the number of all motion episodes; (2) absolute distance, calculated as the cumulative sum of the overall covered distance for all motion episodes; (3) average maximal movement speed, corresponding to the mean of all maximal speeds of second gear episodes; (4) dwell time, i.e., the average time it took the rats to execute a motion episode; (5) spatial spread, i.e., the mean distance covered within a motion episode; (6) curvature of movement tracks, i.e., the ratio of the detected real motion track distance and the distance of the virtual line connecting two coordinate pairs directly during temporal windows of 0.5 s. Curvatures were averaged across all windows of a given motion episode.

Our experimental paradigm allowed for *post-hoc* splitting of the experimental groups into rats monitored after 12 ± 2 days and 28 ± 2 days weeks of recovery. We separately analyzed the correlation between the six behavioral endpoints and the estimated absolute population of TH-positive neurons in the SNc and VTA, the corresponding LI of cell depletion and the observed weight reduction for the 6-OHDA and vehicle injected control group. We utilized bootstrap regressions, which do not rely on a normal distribution of data.

### Statistical analysis

All *post-hoc* analyses were performed using the MATLAB Statistics Toolbox and the BRAVO Toolbox for Bootstrap Regression Analysis of Voxelwise Observations. In case of non-normal data distributions, we employed non-parametric statistical testing (Wilcoxon rank sum test). An alpha level of 0.05 was used for all statistical tests. All statistical results are given as mean ± standard deviation (SD). We corrected *P*-values for multiple comparisons using the false discovery rate (FDR) method (Benjamini and Hochberg, 1995) in case of descriptive behavioral results and multiple bootstrap regression analyses. Bootstrap regressions were performed with 5000 iterations. For descriptive analysis of behavioral endpoints alone, *P*-values were FDR-corrected for a total of 48 different comparisons (2 experimental groups, 6 behavioral endpoints; 4 different comparisons: preoperative vs. 12 + 28 days postoperative, preoperative vs. 12 days postoperative, preoperative vs. 28 days postoperative and 12 days postoperative vs. 28 days postoperative). For bootstrap regressions, *P*-values were FDR-corrected for a total of 55 regressions (the estimated neuronal population of the SNc and VTA, the LI, weight reduction and 6 behavioral endpoints).

## Results

### Stereological counting of TH-positive SNc and VTA neurons

Bilateral injections of 15 μg 6-OHDA into the SNc resulted in extensive cell death of dopaminergic neurons in the SNc and intermediate cell death in the VTA (Figure 1C). The estimated population of TH-positive neurons for combined SNc-ROIs was reduced by −95% in PD rats compared to controls. The absolute number of cells within combined SNc-ROIs was 253.8 ± 196.9 in PD vs. 5098 ± 1189 in control rats (*P* = 0.001). For combined VTA-ROIs, the estimated population of TH-positive neurons was reduced by −51.4% in PD rats compared to controls. Here, the absolute number of cells within combined VTA-ROIs was 3073 ± 766 in PD vs. 6318 ± 828 in control rats (*P* = 0.001). We also tested whether the cell estimates differed significantly between the two subgroups that were monitored 12 and 28 days after lesioning. No statistically significant difference was found for either VTA or SNc-ROIs of 6-OHDA or vehicle injected rats (*P*-values > 0.25, data not shown). Furthermore, the estimated populations within single ROIs did not differ significantly between left vs. right hemispheres in PD and control rats, respectively (*P* > 0.2). Likewise, the LI for 6-OHDA treated rats was 0.14 ± 0.2 and 0.08 ± 0.01 for controls (*P* = 1, data not shown).

### Postsurgical course

Careful daily inspection of PD and control rats in the home cage environment revealed reduced spontaneous locomotion and movement speed of 6-OHDA-treated rats. Body posture appeared with a hunchback-like shape and hind limb rigidity was detectable upon manual assessment. PD rats displayed aphagia and adipsia leading to a reduction in body weight of 19 ± 7.7% of the preoperative weight in PD and 1.5 ± 4.5% in control rats prior to postoperative behavioral monitoring (*P* = 0.002). Three PD rats were euthanized, as they did not exhibit stabilization of weight loss within 12 days after surgery. None of the control rats showed overt movement deficits, aphagia, or adipsia. No additional signs of altered behavior that could indicate persistent pain distress were detected during inspections.

### Circadian activity

**Figure 3:**
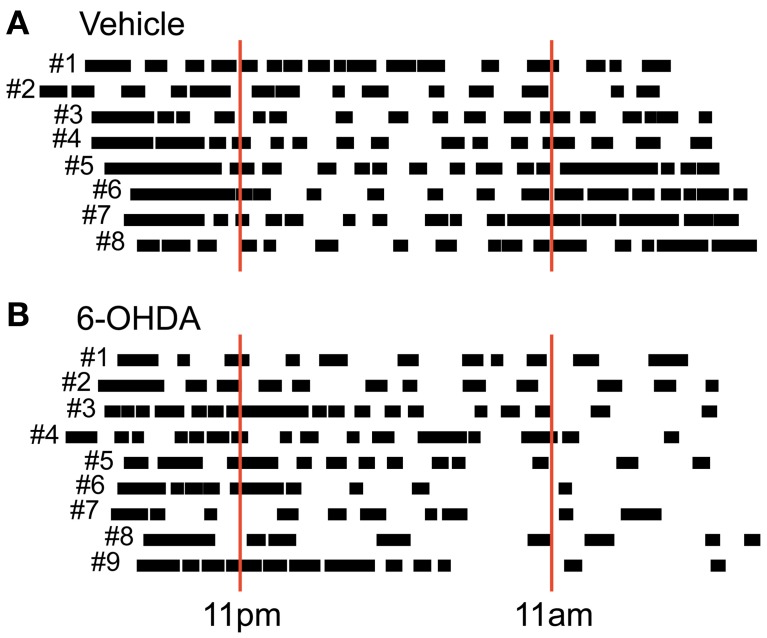
**Circadian post-lesion activity**. Free locomotor activity of all vehicle **(A)** and all 6-OHDA **(B)** injected rats after lesioning. Animal numbers are given left to the respective vertical bars representing global activity phases. Red solid lines mark light switches at 11 pm (lights on) and 11 am (lights off) that would take place under usual housing conditions. Note that behavioral monitoring was performed under constant dark conditions.

Free locomotion monitoring took place under constant dark conditions for at least 23 continuous hours. Although no external light cues were given we saw a pattern of locomotor activity reflecting a circadian rhythm in preoperative (data not shown) and postlesion recordings in vehicle injected controls (Figure 3A). In most rats we found two activity phases that were separated by a phase of reduced activity lasting ~12 hours each. This corresponds to the fact that our rats were accustomed to a 12 hour day/night cycle switching at 11 am (lights off) and 11 pm (lights on). Experiments were usually started between 5 and 6 pm and held under constant dark conditions. In PD rats, however, we found a partially disturbed circadian rhythm (Figure 3B) with two major changes. First, PD rats tended to show a prolonged initial global activity period and less distinguishable transition periods. Second, PD rats did not exhibited a clear rebound of activity in the later phase of monitoring.

### Bimodal distribution of maximal speed values

**Figure 4:**
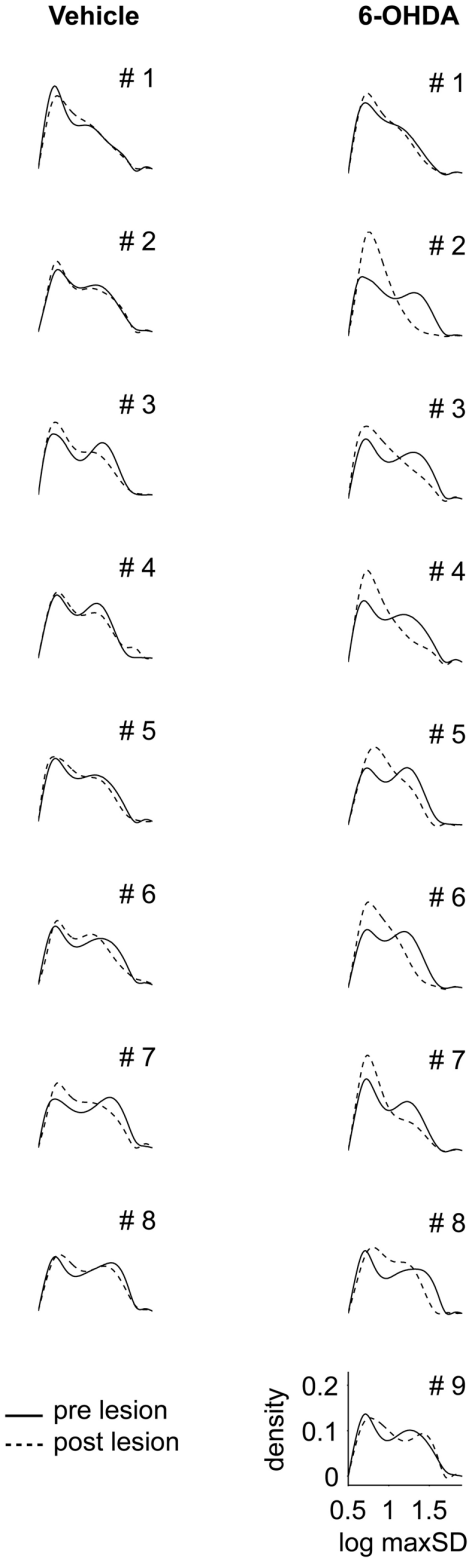
**Distribution of maximal speed values**. Density functions for logarithmic values of maximal speeds (log max-SD) are shown for all control (left column) and all 6-OHDA lesioned rats (right column). Solid and dashed lines denote log max-SD distributions before and after lesioning, respectively. Animal numbers are given in the right top corner. log max-SD, base 10 logarithm of maximal speed in given movement episode; 6-OHDA, 6-hydroxydopamine.

In prelesion recordings, characterization of movement episodes by the log max-SD resulted in a bimodal distribution (Figure 4). In order to distinguish between distinct gears of motion, we fitted a Gaussian mixture model to the empirical distribution of each recording. First gear speeds were centered at a log max-SD of 0.72 ± 0.02 in control and 0.71 ± 0.03 in PD rats, corresponding to a velocity of 5.1 cm/s. Second gear speeds were centered at a log max-SD of 1.24 ± 0.1 in control and 1.24 ± 0.06 in PD rats, i.e., 17.4 cm/s. Notably, 6-OHDA injections resulted in marked changes of the distribution of log max-*SD* values. We observed a partial loss of the bimodal distribution and curve flattening at the center of preoperative second gear episodes. This complicated the fitting of Gaussians and produced spurious results in several animals, e.g., PD rats #1, #2, and #5. Therefore, we separated gears at a log max-SD of 1 (10 cm/s), a value derived from the consistent bimodal distributions of preoperative monitoring.

### Incidence of fast speed movement episodes

**Figure 5:**
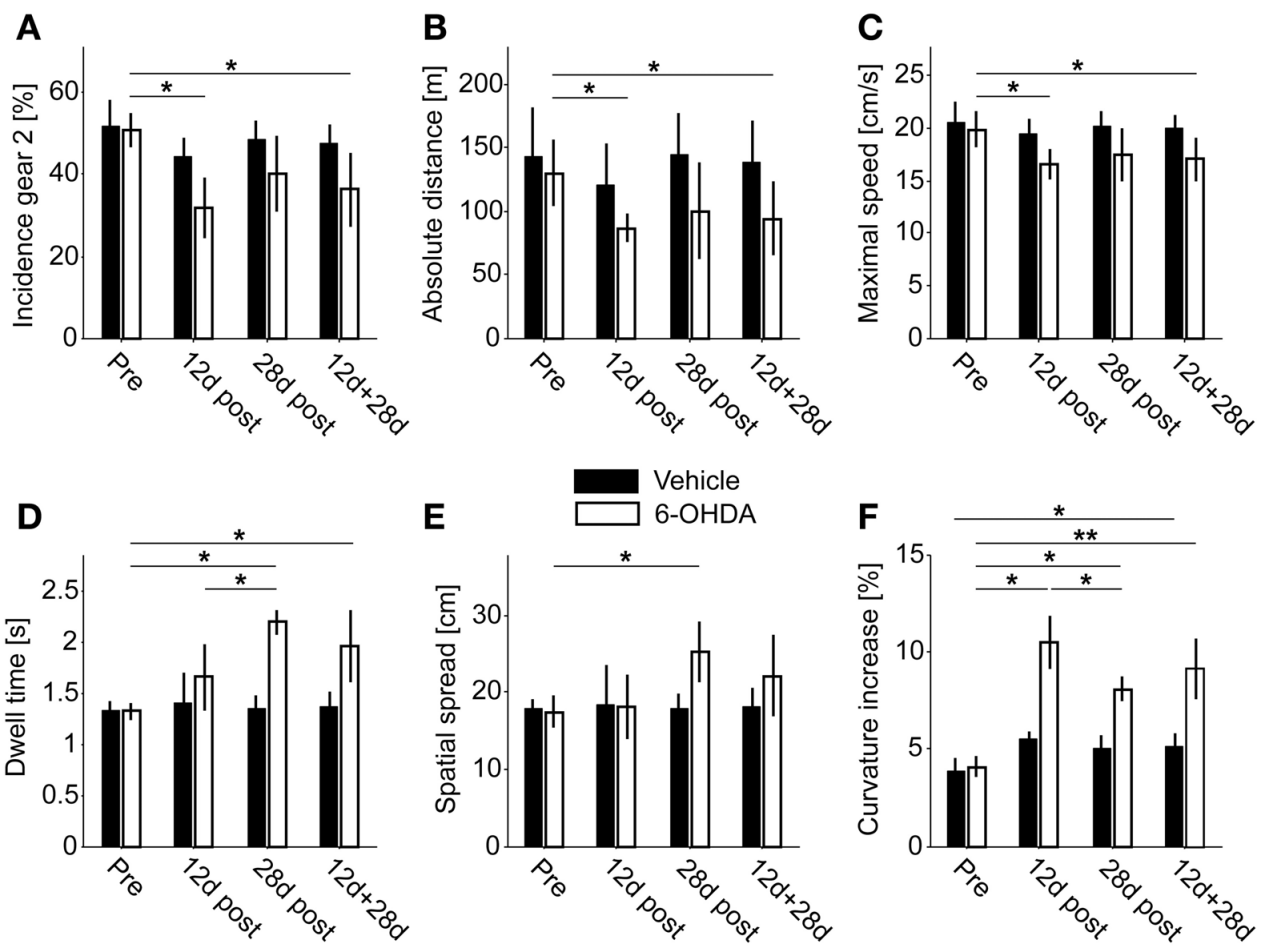
**Behavioral endpoints. (A)** Incidence of second gear episodes. **(B)** Absolute covered distance. **(C)** Average maximal movement speed of second gear episodes. **(D)** Dwell time of second gear episodes. **(E)** Spatial spread of second gear episodes. **(F)** Increase of curvature in second gear episodes. Single asterisks denote *P* ≤ 0.05, double asterisks denote *P* ≤ 0.001 (corrected).

Comparing the number of first gear and second gear episodes per recording, we saw a shift in their incidence after 6-OHDA lesioning (Figure 5A). In the PD group, the incidence of second gear episodes decreased from 50.6 ± 4.1% to 36.4 ± 9% (pre- vs. 12 + 28 days postoperatively, *P* = 0.01, corrected). In vehicle injected controls the proportion of first gear and second gear episodes remained the same (51.8 ± 6.1% vs. 47.4 ± 4.7%, pre- vs. 12 + 28 days postoperatively, *P* = 0.26, corrected). Considering the two subpopulations separately, we only saw a significant difference after 12 days of recovery in PD rats (*P* = 0.02 at 12 days vs. *P* = 0.08 at 28 days; *P* = 0.35 for 12 vs. 28 days; corrected).

### Overall covered distance

6-OHDA injections led to a reduction of the total covered distance within 23 hours of spontaneous locomotion (PD rats: 129.9 ± 25.8 m vs. 94.3 ± 28.7 m, pre- vs. 12 + 28 days postoperatively, *P* = 0.05; control rats: 143.2 ± 38 m vs. 138.9 ± 31.7 m, pre- vs. 12 + 28 days postoperatively, *P* = 1, corrected; Figure 5B). Again, this difference was significant for the PD group at 12 days, but not at 28 days of recovery (*P* = 0.02 at 12 days vs. *P* = 0.35 at 28 days; *P* = 1 for 12 vs. 28 days; corrected).

### Average maximal movement speed

The log max-SD across all second gear episodes of full motion was significantly reduced in PD rats (19.9 ± 1.7 cm/s vs. 17 ± 2 cm/s, pre- vs. 12 +28 days postoperatively, *P* = 0.02; control rats: 20.6 ± 1.8 cm/s vs. 20 ± 1.3 cm/s, pre- vs. 12 +28 days postoperatively, *P* = 1, corrected; Figure 5C). Likewise, the difference was significant also within the PD group at 12 days of recovery (*P* = 0.02 at 12 days vs. *P* = 0.18 at 28 days; *P* = 0.92 for 12 vs. 28 days; corrected). We found no significant difference for first gear episodes (*P* = 0.07 pre- vs. 12 + 28 days postoperatively, *P* = 0.25 at 12 days, *P* = 0.09 at 28 days; *P* = 0.26 for 12 vs. 28 days; corrected).

### Dwell time

The average dwell time of second gear episodes was increased in lesioned animals but not in controls (PD rats: 1.32 ± 0.08 s vs. 1.96 ± 0.35 s, pre- vs. 12 + 28 days postoperatively, *P* = 0.01; control rats: 1.33 ± 0.09 s vs. 1.37 ± 0.15 s, pre- vs. 12 + 28 days postoperatively, *P* = 0.84, corrected; Figure 5D). Interestingly, the difference in dwell time was also significant in the 28 days subset of PD rats, but not at 12 days post-surgery (*P* = 0.17 at 12 days vs. *P* = 0.01 at 28 days; *P* = 0.05 for 12 vs. 28 days; corrected). Furthermore, the dwell time of first gear episodes was also significantly prolonged (*P* = 0.001 pre- vs. 12 + 28 days postoperatively, *P* = 0.01 at 12 days, *P* = 0.008 at 28 days; *P* = 0.98 for 12 vs. 28 days; corrected).

### Spatial spread

The spatial spread accomplished within a given episode (averaged across all second gear episodes of full motion) was increased after 6-OHDA lesioning (PD rats: 17.5 ± 2 cm vs. 22.1 ± 5.3 cm, pre- vs. 12 + 28 days postoperatively, *P* = 0.08; control rats: 18 ± 1.2 cm vs. 18 ± 2.6 cm, pre- vs. postoperatively, *P* = 0.83, corrected; Figure 5E). Comparable to the dwell time, the difference was significant at 28 days in PD-rats, but not at 12 days or within the combined group (*P* = 1 at 12 days vs. *P* = 0.01 at 28 days; *P* = 0.08 for 12 vs. 28 days; corrected). Similar to the dwell time, the spatial spread was also significantly enlarged for first gear episodes (*P* = 0.001 pre- vs. 12 + 28 days postoperatively, *P* = 0.02 at 12 days, *P* = 0.008 at 28 days; *P* = 0.98 for 12 vs. 28 days; corrected).

### Curvature

6-OHDA injections led to an increase of the mean curvature of motion tracks (PD-rats: 4.07 ± 0.52% vs. 9.15 ± 1.66%, pre- vs. 12 + 28 days postoperatively, *P* = 0.002). Notably, the curvature was also significantly increased in vehicle injected control rats, but only for the comparison of pre vs. combined postoperative groups (3.850 ± 0.70% vs. 5.15 ± 0.64%, pre- vs. 12 + 28 days postoperatively, *P* = 0.02, corrected; Figure 5F). However, in PD rats we saw a stronger increase 12 days after injections (*P* = 0.02 at 12 days) and a significant recovery of curvature ratios 2 weeks later (*P* = 0.05 for 12 vs. 28 days). At the later time point the ratio was still significantly enhanced (*P* = 0.01 at 28 days). The same pattern of statistically significant differences was observed for first gear episodes (*P* = 0.001 pre- vs. 12 + 28 days postoperatively, *P* = 0.01 at 12 days, *P* = 0.008 at 28 days; *P* = 0.05 for 12 vs. 28 days; corrected).

### Bootstrap regressions

**Figure 6:**
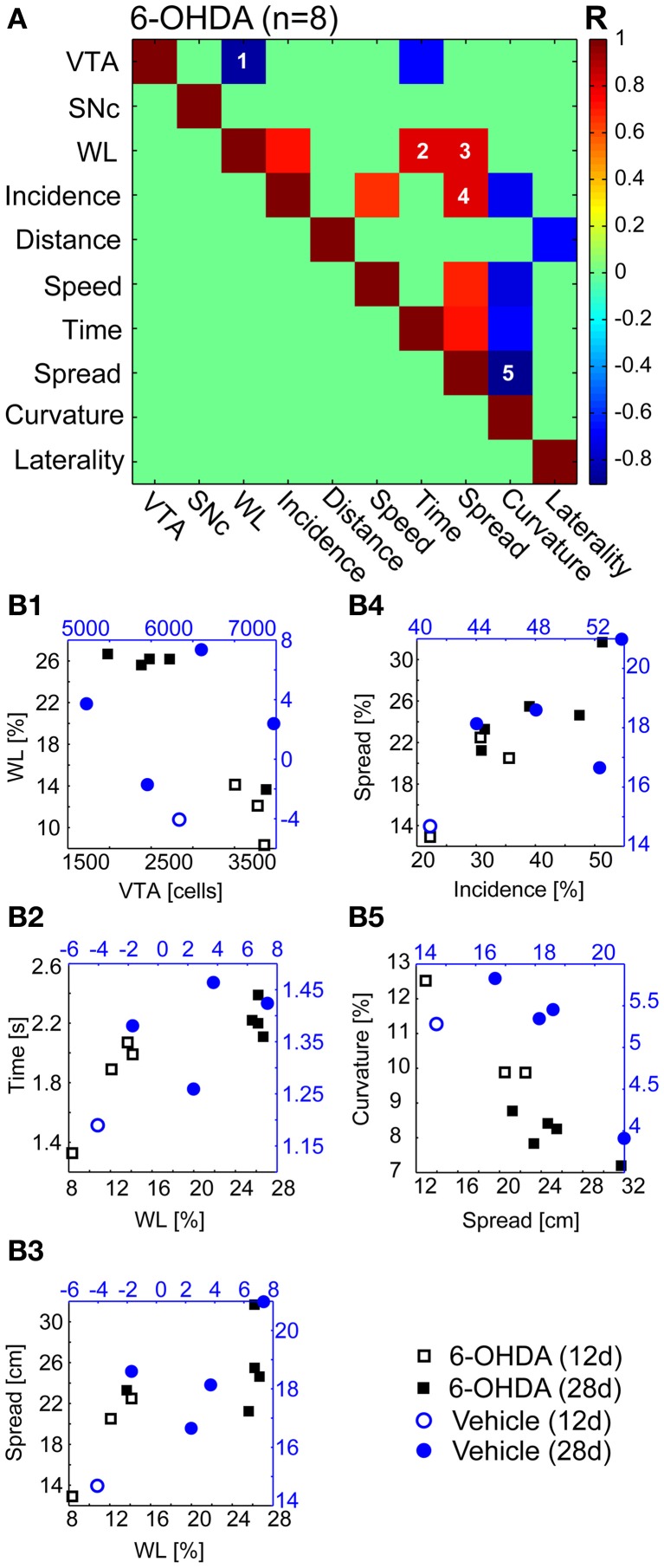
**Bootstrap regressions. (A)** Independent bootstrap regressions (*n* = 55) of the 6-OHDA group (*n* = 8 animals). The bootstrapping *R*-value is color-coded and clipped for correlations with *P* > 0.1 after FDR-correction (green fields). **(B1–5)** Correlations with *P* < 0.05 after FDR-correction. Open and filled markers indicate rats from 12 to 28 days subsets, respectively. 6-OHDA, 6-hydroxydopamine.

We found a significant correlation for five of 55 independent comparisons (Figure 6A) in 6-OHDA treated rats (*n* = 8). The estimated number of VTA, but not of SNc neurons correlated negatively with the magnitude of weight reduction (*R* = −0.86, Figure 6B1). Weight loss also correlated with dwell time (*R* = 0.8, Figure 6B2) and spatial spread (*R* = 0.8, Figure 6B3). Spatial spread also correlated with the incidence of second gear episodes (*R* = 0.71, Figure 6B4). Furthermore, the curvature of motion tracks obtained postoperatively correlated negatively with the spatial spread (*R* = −0.9, Figure 6B5). Further 9 comparisons were found statistically significant before FDR-correction and yielded *P*-values < 0.1 after correction (Figure 6A). Among them was the only comparison, incidence of second gear episodes vs. maximal speed, that was also found highly correlated in vehicle injected controls (*R* = 1, data not shown).

## Discussion

The main findings of our study are that the stereotypical locomotion pattern of rats exhibiting a first and a second gear of motion is partially lost after bilateral dopaminergic denervation. The reduced incidence of second gear episodes points at distinct deficits in the execution of fast motor sequences. However, although generally slowed, bilaterally lesioned rats also displayed bouts of spontaneous locomotion, to some degree similar to kinesia paradoxa observed in PD patients. Contrary to expectation, we observed an abnormally increased spatial spread and increased motion time during episodes of full motion in lesioned rats. Moreover, we noted larger curvature values in movement paths of lesioned rats. Upon acceleration, curvature values showed a reduction toward physiological values. All these changes were accompanied by an alteration of circadian locomotor activity.

The depletion of estimated dopaminergic cell populations in the SNc and VTA in our disease model was comparable to that observed in humans suffering from advanced PD, where the VTA is known to be significantly less depleted than the SNc (Hirsch et al., 1988). However, the mesocorticolimbic dopamine projections are vulnerable in advanced PD as well, and seem to contribute to the complex clinical picture encountered in humans, especially at later disease stages (Thobois et al., 2010). It is worth mentioning that our stereological results were similar to cell-loss estimates of 40–50% in the VTA described in human postmortem studies (Uhl et al., 1985; Hirsch et al., 1988; Dymecki et al., 1996; McRitchie et al., 1997; Thobois et al., 2010). The estimated number of SNc neurons of vehicle injected rats in our study was comparable to the number of TH-positive cells reported by a previous study (Fox et al., 2001) that investigated Fisher 344 × Brown Norway hybrids. Other studies reported significantly higher TH-positive cell counts in different rat strains (Lewis rats, Strackx et al., 2008; Long-Evans rats Healy-Stoffel et al., 2012; Sprague-Dawley rats, Walker et al., 2012) suggesting large differences in the absolute number of midbrain dopaminergic cells in different rat strains.

PD rats in our study displayed a considerable reduction of body weight (~20%). Weight loss and associated metabolic changes might by themselves have an influence on locomotion. We included the reduction of body weight into multiple bootstrap regressions and investigated the interdependence between behavioral endpoints, dopaminergic cell loss and weight reduction. We found a significant negative correlation between the VTA, but not the SNc dopaminergic population, and the magnitude of weight loss. Thus, abnormal feeding behavior and more generally abulia, was related to cell loss within the mesolimbic dopaminergic system in our study (Redgrave et al., 2010).

Our analysis approach allowed the discrimination of naturally occurring motion episodes in severely dopamine depleted and overtly akinetic and bradykinetic rats. The characterization of motion episodes by the maximal rather than the average speed accomplished in a given episode enabled us to analyze rare and, given the pronounced parkinsonian phenotype of our rats, unexpected episodes of qualitatively altered motor activity. These motion episodes were still characterized by a reduced incidence (i.e., poverty of movement or akinesia), reduced overall traveled distance (i.e., hypokinesia) and reduced maximal speed (i.e., bradykinesia) compared to controls. Hence, important hallmark symptoms of PD were clearly detectable in motion episodes of 6-OHDA rats. However, we did not see a significant correlation of these three behavioral endpoints with the estimated populations of the SNc and VTA.

Overall, the quantitative change in traveled distance was rather small. A disruption of the circadian rhythm, a cardinal non-motor symptom in human PD patients (Videnovic and Golombek, 2013) and present in animal models of PD (Kudo et al., 2011; Willison et al., 2013) may introduce a bias. Higher absolute activity values may result from prolongation and fragmentation of activity and reduced sleep phases. Our analysis method could not differentiate between awake resting and true sleep, but conclusions could still be drawn from the overall circadian activity pattern. Here, we observed a more fragmented pattern of activity together with a nearly absent second activity phase. Moreover, PD rats showed a significantly reduced absolute number of motion episodes in comparison to controls (data not shown). Together, this argues against a circadian bias toward higher distance values in our data.

The finding of increased dwell times and spatial spread in first and second gear motion episodes of PD rats was rather unexpected. The spatial spread in the 6-OHDA group was increased to values not seen in any pre-lesion or control recordings. Theoretically, an increased dwell time could be explained as a function of slowed locomotion. To reach the same location, bradykinetic rats may simply need more time. This interdependence should theoretically manifest in a negative correlation between speed and dwell time or spatial spread. To the contrary, our data revealed a positive correlation between speed and spatial spread, albeit not significant after FDR-correction. That is, faster PD rats showed spatially extended second gear episodes. Could a postlesion increase in curvature explain increased dwell times? Arguing against that, curvature values were significantly negatively correlated with spatial spread, and also with dwell time, speed and incidence of second gear episodes (before FDR-correction). Furthermore, a higher incidence of second gear behavior correlated significantly with prolonged spatial spread. Such an increased incidence could be the result of an uneven effect of 6-OHDA lesioning and stronger diminishment of first gear in comparison to second gear activity. This would also be supported by the finding that 6-OHDA induced weight loss correlated positively with the incidence of second gear activity (before FDR-correction). Taken together, increased spatial spread and dwell times constitute an abnormal behavioral characteristic of PD rats.

An abnormal increase of locomotor activity in bilaterally lesioned rats has been described in response to a pharmacological challenge (Schallert et al., 1978). Unexpected bursts of locomotion in otherwise akinetic patients are also well known to occur in selected PD patients or cases with postencephalitic parkinsonism (kinesia paradoxa). The sudden ability of our rats to cover longer distances within a motion episode may thus represent a rat equivalent of this condition. There are, however, important differences to classical kinesia paradoxa, which is often triggered by external sensory cues (Martin, 1967).

One possible explanation for prolonged dwell times and increased spatial spread could be problems with the termination of movement. In PD patients, deficiency in smooth motion termination is known (Dounskaia et al., 2009), along with an inability to promptly change generated force or quickly re-plan current motion. Typically, PD patients show an asymmetric evolution of velocities during the execution of goal-directed behaviors with initial fast accelerations (Flash et al., 1992). In our data we found a symmetric evolution of acceleration between PD and control rats. That is, we saw a linear relationship between the dwell time and the time point where PD or control rats accomplished their maximal speed in a given episode (data not shown). Thus, the observed behavioral abnormalities do not support the presence of the hastening phenomenon (unwanted acceleration of movement in human PD patients) in our rats. Finally, it is also conceivable that the abnormal drive to continue exploration or food search could be a behavioral consequence of metabolic changes accompanying weight loss (Redgrave et al., 2010).

Another abnormal feature was found in the movement path's curvature. The ratio between real and direct distance of first and second gear motion episodes was significantly enlarged in PD rats and, to a lesser extent, in controls. What could be the cause of increased curvatures? Unilaterally depleted rats display a spontaneous ipsiversive motor bias (Ungerstedt, 1971). Hence, curvature increases could result from spontaneous partial turning behavior provoked by asymmetric dopaminergic lesioning in our rats. However, the calculated LI did not correlate with curvature or any other behavioral endpoint except distance (before FDR-correction). Furthermore, unilateral 6-OHDA lesions were shown to shorten steps in spontaneous walking (Metz et al., 2005), thus modeling the shuffling gait of PD patients (Knutsson, 1972). Hind limb rigidity was detected upon manual assessment in our PD rats and could have contributed to disturbed locomotor patterns with reduced step sizes, axial instability and loss of balance during walking. Hemiparkinsonian rats, when tested by e.g., beam-walking, also exhibit difficulties in motor coordination (Truong et al., 2006). Interestingly, we found a significant negative correlation between curvature and spatial spread, as well as maximal speed, dwell time and incidence of second gear activity (before FDR-correction). Thus, faster running or greater spreads were associated with straighter movement paths in PD rats. If limb rigidity was indeed related to the expression of increased curvature values, then the ability of PD rats to generate fast motion with reduced curvature values may reflect an overcoming of rigidity for brief periods.

We saw some differences between lesioned animals that were monitored at an early (12 days) and later stage (28 days) after 6-OHDA lesioning. Most strikingly, the dwell time was significantly longer at 28 days in comparison with 12 days postlesion. Contrary, curvature increases significantly decreased again at the later point of investigation. Importantly, no significant difference in cell counts was observed between the two subsets. It remains difficult to infer which compensatory mechanisms were at work here. The milder but still significant increase of curvature in controls, in conjunction with the significant decrease of curvature values in PD rats with time, could argue for an influence of and recovery from surgery *per se*. The emergence of an abnormally increased behavior such as kinesia paradoxa may have in turn developed over time when the rats recovered fully from surgery. Despite putative effects of recovery from surgery, absolute distance, speed and incidence of second gear activity were comparably decreased at an early and later point of investigation.

We conclude that long-term behavioral observations of spontaneous locomotion offers new perspectives on distinctly different modes of motion in a rat model of advanced PD. The present behavioral analysis, in conjunction with *in-vivo* electrophysiology, may be particularly suited to reveal neural mechanisms underlying motor fluctuations such as kinesia paradoxa, and may provide further insights into the complex pathophysiology of PD.

## Author contributions

Benjamin Grieb, Gerhard Engler, Ismini Papageorgiou, Wolfgang Hamel, and Christian K. Moll designed research; Benjamin Grieb, Gerhard Engler, and Ismini Papageorgiou performed experiments; Benjamin Grieb, Constantin von Nicolai, Andrew Sharott, and Ismini Papageorgiou analyzed the data; Benjamin Grieb, Constantin von Nicolai, Andrew Sharott, Andreas K. Engel, and Christian K. Moll wrote the manuscript.

### Conflict of interest statement

The authors declare that the research was conducted in the absence of any commercial or financial relationships that could be construed as a potential conflict of interest.
